# Clinical characteristics of pancreatic and biliary tract cancers in Lynch syndrome: A retrospective analysis from the Finnish National Lynch Syndrome Research Registry

**DOI:** 10.3389/fonc.2023.1123901

**Published:** 2023-02-01

**Authors:** Kristina Zalevskaja, Jukka-Pekka Mecklin, Toni T. Seppälä

**Affiliations:** ^1^ Applied Tumor Genomics Research Program, Research Programs Unit, University of Helsinki, Helsinki, Finland; ^2^ Department of Gastrointestinal Surgery, Helsinki University Central Hospital, Helsinki, Finland; ^3^ Faculty of Sport and Health Sciences, University of Jyväskylä, Jyväskylä, Finland; ^4^ Department of Education and Research, Jyväskylä Hospital Nova, Jyväskylä, Finland; ^5^ Faculty of Medicine and Health Technology and Tays Cancer Centre, University of Tampere, Tampere, Finland

**Keywords:** Lynch syndrome (LS), hereditary nonpolyposis colon cancer (HNPCC), pancreatic cancer, biliary tract cancer, microsatellite instability (MSI)

## Abstract

**Introduction:**

Patients with Lynch syndrome (LS) have an increased lifetime risk of pancreatic cancer (PC) and biliary tract cancer (BTC). These cancers have a notoriously pessimistic prognosis due to late diagnosis and limited therapeutic options. There are limited data based on small cohorts reviewing PC and BTC in LS patients.

**Methods:**

In this retrospective study of the Lynch Syndrome Registry of Finland (LSRFi), records of genetically verified LS patients diagnosed with PC or BTC between 1982 and 2020 were analyzed.

**Results:**

Thirty-nine patients were included: tumor(s) were in the pancreas in 26 patients, in the biliary tract in 10, and in the ampulla of Vater in three. A pathogenic germline variant was found in *MLH1* in 33 of 39 patients. Twenty-six patients with 28 tumors located in the pancreas were identified: 23 pancreatic ductal adenocarcinomas (PDACs) and five neuroendocrine tumors (NETs). The median age at diagnosis of PC was 64 years (range of 38–81). In PC, the 5-year overall survival (OS) rate was 20%, and in PDAC, it was 13.6%. Ten patients with BTC were diagnosed: two intrahepatic, five perihilar, two distal extrahepatic cholangiocarcinomas, and one gallbladder carcinoma. Eight patients were male, and the median age at diagnosis was 54 years (range of 34–82). The 5-year OS rate for BTC was 30%. Metachronous tumors were diagnosed in 28 patients (70%). Colorectal cancer was the most common metachronous tumor, diagnosed in 20 patients (51%), and diagnosed prior to PC or BTC in all cases. Curative surgery was attempted on 17 of 39 patients. For 30 patients (91%), the cause of death was PC or BTC; two patients died from another LS-associated cancer, and one died from a stroke.

**Conclusion:**

Although the survival of LS patients with PC or BTC is better than in sporadic cancers, it is still poor and may be reflected by the relatively higher surgical resectability accounted for by the earlier age of onset. More studies on analyses of the molecular and immune profile, screening, and management of LS-associated pancreaticobiliary cancers are warranted.

## 1 Introduction

Lynch syndrome (LS), previously known as hereditary nonpolyposis colorectal cancer (HNPCC), is an autosomal dominant disorder caused by pathogenic germline variants in one of the DNA mismatch repair (MMR) genes, *MLH1*, *MSH2*, *MSH6*, or *PMS2*, or by deletions in the *EPCAM* gene (1–3). It is the most common hereditary cancer syndrome, with a prevalence estimated as high as 1 in 279 (4). Pathogenic MMR variant carriers have a high lifetime risk of developing colorectal and endometrial cancers and an increased risk of developing gastric, ovarian, urothelial, pancreatic, biliary tract, small bowel, prostate, breast, brain, and skin cancers, depending on the gene affected (5). LS-associated cancers usually display MMR deficiency (dMMR) that leads to microsatellite instability (MSI) in the tumors.

Increased risk of pancreatic cancer (PC) in LS carriers was first observed by Lynch et al. in 1985 (6). In 1992, Mecklin et al. described 11 LS patients with biliary tract cancer (BTC), suggesting an association between BTC and LS (7). Since then, numerous retrospective studies and one review have confirmed an increased incidence of PCs and BTCs in LS patients (8–12). The Prospective Lynch Syndrome Database (PLSD) report has shown different lifetime risks for PC depending on the germline mutation variant (5).

LS-associated colorectal, gynecological, and gastric cancers have a better prognosis than sporadic cancers (5, 13–16). Unfortunately, PC and BTC remain aggressive and have a poor prognosis. As of lately, immune checkpoint inhibitor therapy has been an exciting development in the treatment of solid tumors with MSI and dMMR, including PC and BTC, with promising results (17–20). However, there are limited data based only on small cohorts reviewing pancreatic and biliary tract malignancies in LS patients.

In this article, we present the largest cohort of LS patients with PC and BTC to date and characterize their clinical features.

## 2 Methods

### 2.1 Study cohort

We retrospectively reviewed the medical records of patients in the Lynch Syndrome Registry of Finland (LSRFi) who were diagnosed with pancreatic or biliary tract malignant tumors between 1982 and 2020. The nationwide registry, established in 1982, includes, at present, 1,800 verified pathogenic variant carriers from 400 families and contains clinicopathological information on all cancers of registered individuals. The data have been regularly cross-checked against the Finnish national cancer registry.

This multicenter retrospective study was approved by the national authority for registry research (Findata), waiving the requirement for informed consent to use data obtained from medical records.

### 2.2 Survival analyses

Overall survival (OS) was defined as the time from diagnosis until death from any cause or the last date of confirmed survival. OS was analyzed in R using the Kaplan–Meier method (21).

### 2.3 Pathological classification

Pancreatic adenocarcinomas (PDACs) were graded according to histopathological WHO criteria (22). Pancreatic neuroendocrine tumors (NETs) were classified according to the WHO 2020 classification for pancreatic neuroendocrine neoplasms (23). Biliary tract tumors were classified according to system based on their anatomical location and categorized as intrahepatic, perihilar, or distal cholangiocarcinomas (24). According to the WHO classification of digestive system tumors, adenocarcinomas of the ampulla of Vater are histologically closer to the small intestine but anatomically near the pancreas and biliary tract (22). Therefore, this rare type of cancer was included in the cohort but analyzed separately.

## 3 Results

### 3.1 Patient characteristics

**Figure 1:**
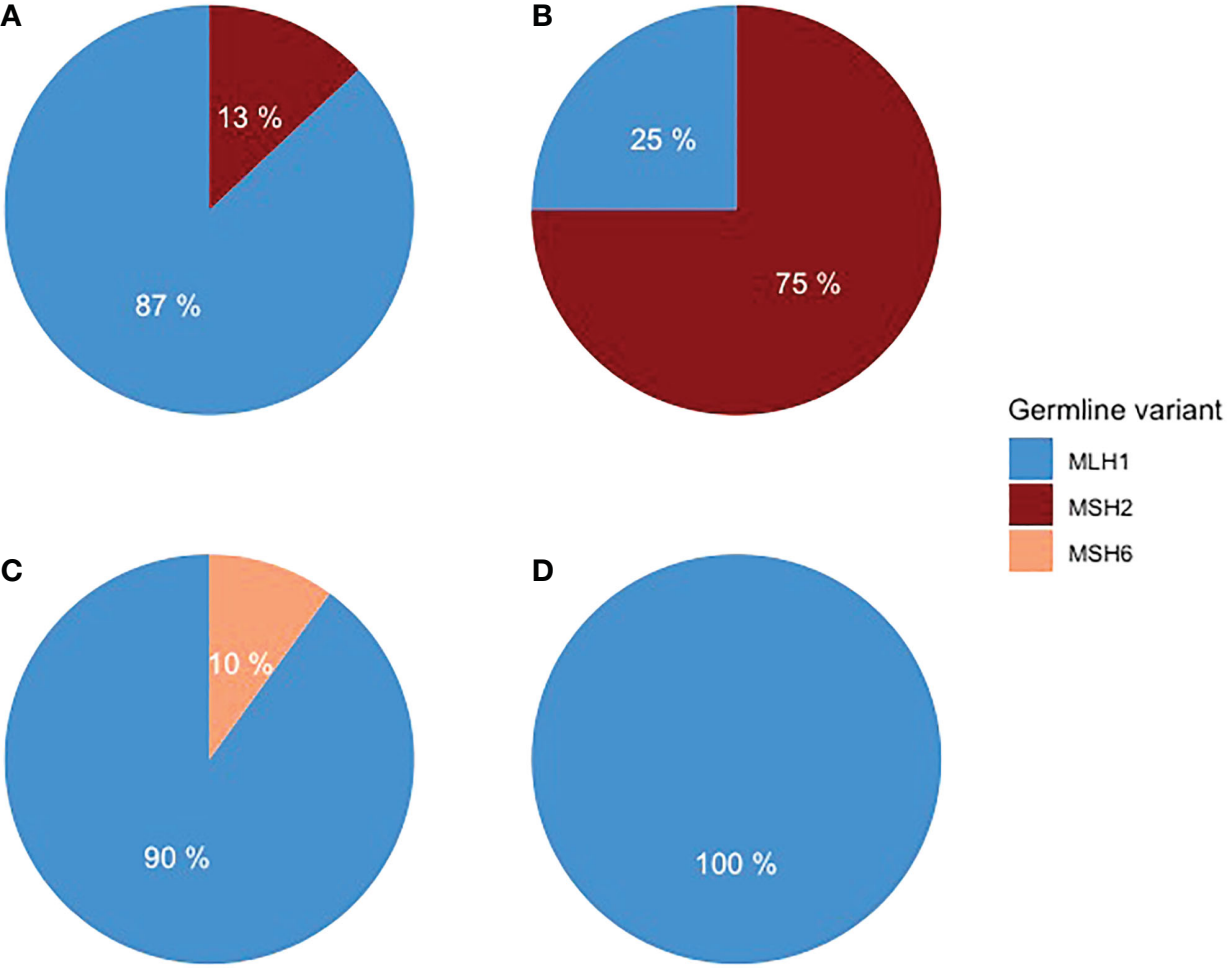
Distribution of germline variants in **(A)** PDAC, **(B)** pancreatic NET, **(C)** BTC, and **(D)** ampullary adenocarcinoma.

Forty LS patients were diagnosed with PC or BTC or ampullary cancer between 1982 and 2020 (**Table 1**). Among them, tumors in 26 patients were in the pancreas, 10 in the biliary tract, and three in the ampulla of Vater. One patient was excluded from the study due to non-pancreatic and non-biliary histology. A pathogenic germline variant of *MLH1* was detected in 33 patients, *MSH2* in five, and *MSH6* in one patient. *MLH1* variant carriers had 21 out of 26 PCs, nine out of 10 BTC, and all three ampullary cancers (**Figure 1**).

**Table 1:** Clinicopathological characteristics of PC, BTC, and ampullary cancer in LS patients.

Cancer type	Age	Sex	Family id	LS patients in the family	Primary site	Histology	Operation	Chemotherapy	Radiation therapy	Metachronous tumors	Gene	OS (month)
Ampulla of Vater	65	F	4	4	Ampulla of Vater	Ampullary adenocarcinoma	R0	no	no	CRC×3, uterus, ovary, ventricle	*MLH1*	27
Ampulla of Vater	53	F	1	31	Ampulla of Vater	Ampullary adenocarcinoma	R0	yes	yes		*MLH1*	32
Ampulla of Vater	49	M	87	10	Ampulla of Vater	Ampullary adenocarcinoma	R0	NA	NA	CRC×2, prostate	*MLH1*	275
Biliary tract	48	F	73	24	Gallbladder	Carcinoma of gallbladder	R0	no	no		*MLH1*	Alive
Biliary tract	53	M	19	7	Common bile duct	Cholangiocarcinoma	palliative	no	yes	CRC	*MLH1*	6
Biliary tract	80	F	231	7	Common bile duct	Cholangiocarcinoma	R0	NA	NA	Uterus, ureter	*MSH6*	Alive
Biliary tract	50	M	195	6	Intrahepatic	Cholangiocarcinoma	no	yes	no		*MLH1*	9
Biliary tract	68	M	205	4	Intrahepatic	Cholangiocarcinoma	R0	no	no	CRC×4	*MLH1*	Alive
Biliary tract	55	M	1	31	Perihilar	Cholangiocarcinoma	no	no	no	CRC	*MLH1*	31
Biliary tract	82	M	160	4	Perihilar	Cholangiocarcinoma	no	no	no	CRC×2	*MLH1*	15
Biliary tract	40	M	99	7	Perihilar	Cholangiocarcinoma	no	yes	no	CRC	*MLH1*	9
Biliary tract	34	M	50	34	Perihilar	Cholangiocarcinoma	R1	yes	no		*MLH1*	27
Biliary tract	74	M	61	21	Perihilar	Cholangiocarcinoma	no	yes	yes	CRC	*MLH1*	11
Pancreas	47	M	94	3	head	Ductal adenocarcinoma	no	yes	no		*MLH1*	12
Pancreas	81	F	78	7	head	Ductal adenocarcinoma	no	yes	no		*MLH1*	9
Pancreas	58	M	2	37	head	Ductal adenocarcinoma	palliative	no	no	CRC×2	*MLH1*	1
Pancreas	69	M	9	6	head	Ductal adenocarcinoma	R0	no	no	CRC, ureter	*MLH1*	1
Pancreas	54	F	157	2	head	Ductal adenocarcinoma	no	yes	yes	CRC, uterus	*MLH1*	6
Pancreas	67	F	2	37	head	Ductal adenocarcinoma	R1	yes	no	Uterus, ovary	*MLH1*	Alive
Pancreas	61	M	152	3	head	Ductal adenocarcinoma	R0	no	no	Spinal cord	*MLH1*	129
Pancreas	64	F	54	29	head	Ductal adenocarcinoma	no	no	no	CRC×2, ovary	*MLH1*	3
Pancreas	69	M	38	12	head	Ductal adenocarcinoma	no	yes	no		MSH2	7
Pancreas	68	M	1	31	head	Ductal adenocarcinoma	R0	no	no	CRC, prostate	*MLH1*	8
Pancreas	74	F	112	21	head	Ductal adenocarcinoma	no	no	no	CRC×2, uterus	*MLH1*	6
Pancreas	70	F	24	2	head	Ductal adenocarcinoma	palliative	no	no	Ureter, acusticus neurinoma	*MLH1*	2
Pancreas	86	F	241	4	body	Ductal adenocarcinoma	no	no	no	CRC, uterus	*MLH1*	0
Pancreas	60	M	82	12	body	Ductal adenocarcinoma	R1	yes	no		*MLH1*	32
Pancreas	45	F	98	11	body	Ductal adenocarcinoma	no	yes	no	CRC	*MLH1*	36
Pancreas	80	F	105	10	body	Ductal adenocarcinoma	palliative	yes	no	CRC×2, uterus	*MLH1*	4
Pancreas	53	F	122	3	body	Ductal adenocarcinoma	no	yes	no	Cervix	MSH2	7
Pancreas	71	F	138	2	body	Ductal adenocarcinoma	R0	no	no	Uterus	*MLH1*	Alive
Pancreas	54	F	191	2	body	Ductal adenocarcinoma, NET	R1	yes	no	Breast	MSH2	6
Pancreas	39	F	23	6	tail	Ductal adenocarcinoma	R1	yes	no	Ovary, ventricle	*MLH1*	5
Pancreas	66	M	19	7	tail	Ductal adenocarcinoma	no	no	no	CRC	*MLH1*	1
Pancreas	56	M	10	11	tail	Ductal adenocarcinoma	palliative	no	yes	CRCx2	*MLH1*	15
Pancreas	64	M	146	4	NA	Ductal adenocarcinoma	no	no	no		*MLH1*	3
Pancreas	63	F	1	31	body	NET	no	yes	no		*MLH1*	2
Pancreas	38	F	191	2	body, tail	NETx2	R0	no	no	CRC	*MSH2*	101
Pancreas	69	M	38	12	tail	NET	R1	yes	no		*MSH2*	Alive

NA, not available; CRC, colorectal cancer; NET, neuroendocrine tumor; R0, negative resection margin; R1, positive resection margin; OS, overall survival.

LS was diagnosed prior to PC or BTC in 22 patients and simultaneously in 14 patients. Each patient’s family in LSRFi and the number of LS-diagnosed patients in this family are presented in **Table 1**. Five families had more than one family member diagnosed with PC or BTC.

In 26 patients with 28 tumors located in the pancreas, 23 were PDAC and five were NETs. Fifteen were female, and the median age at diagnosis was 64 years (range of 38–81). The distribution of anatomical locations of PC was head 12, body 10, and tail five (one location was not recorded). Germline variants of *MLH1* were detected in 21 patients and *MSH2* in five patients. One patient had simultaneously PDAC and NET, and one patient had two NETs. In both patients, multiple endocrine neoplasia type 1 (MEN1) syndrome was additionally diagnosed. The diagnosis of pancreatic malignancies in 20 patients was based on clinical, radiological, and pathology reports, but in six patients, it was based on clinical and radiological findings only.

Of the 10 BTC patients, two were intrahepatic, five perihilar, two distal extrahepatic cholangiocarcinomas, and one gallbladder carcinoma. Eight were male, and the median age at diagnosis was 54 years (ranging from 34 to 82). Germline variants of *MLH1* were detected in nine patients and of *MSH6* in one patient. The BTC diagnosis was based on a pathology report in seven patients and on computer tomography in three patients. The patient with gallbladder carcinoma was primarily operated on due to pain caused by gallstones but received an unexpected diagnosis of gallbladder carcinoma. The treatment was later completed with liver segment II–IV resections with R0 margins.

Adenocarcinoma of the ampulla of Vater was diagnosed in three patients, all *MLH1* carriers. Two were women, and the median age at diagnosis was 53 years (ranging from 49 to 65). As all three patients underwent surgery, the diagnosis was verified by a pathological report.

### 3.2 Metachronous tumors

Metachronous tumors were diagnosed in 28 (72%) patients (median number 1; range of 0–6). Colorectal cancer was the most common, diagnosed in 20 patients, and endometrial cancer in eight patients. In all cases, colorectal cancer and endometrial cancer were diagnosed prior to PC or BTC.

### 3.3 Treatment and survival

**Figure 2:**
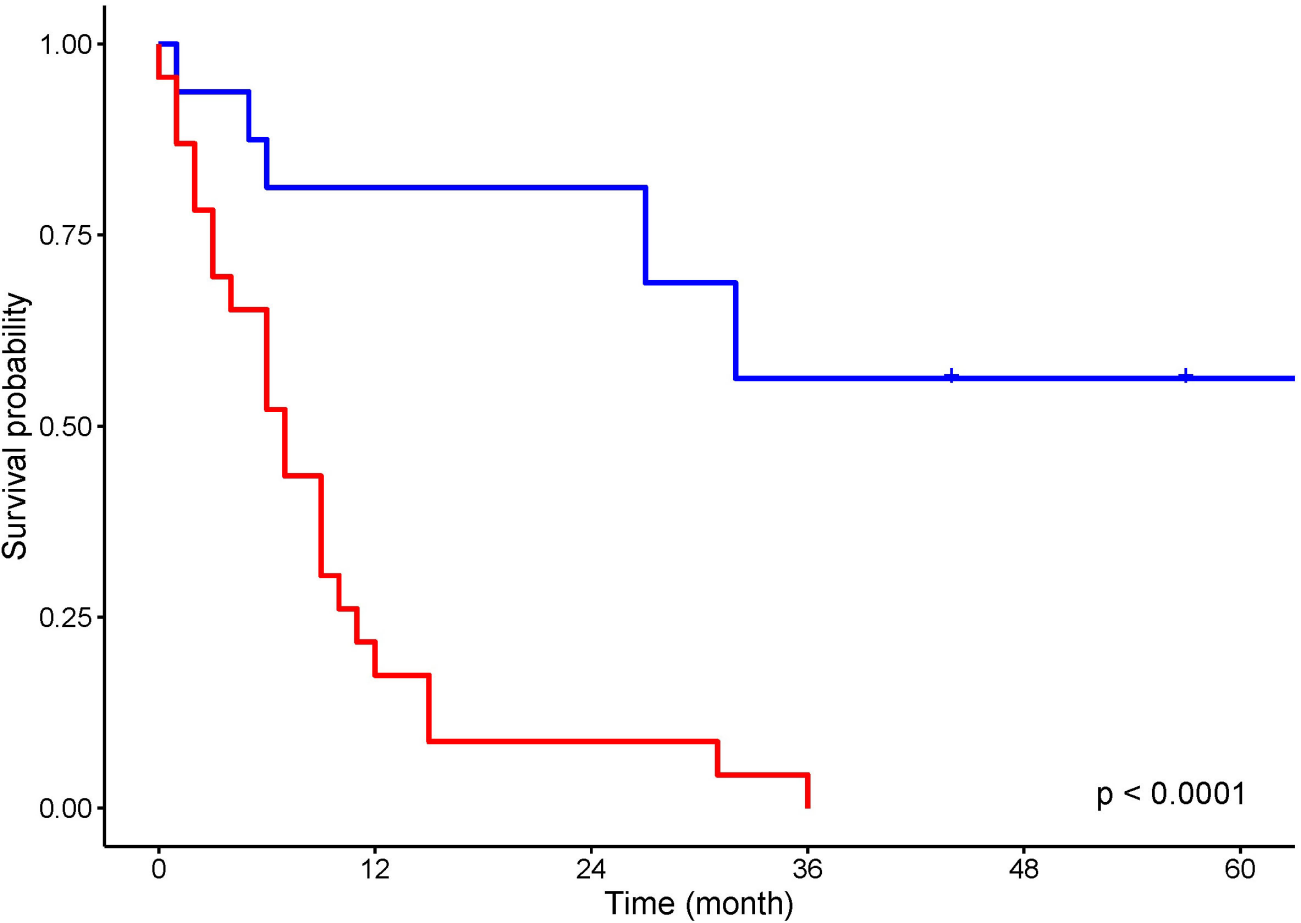
Kaplan–Meier analysis for overall survival of patients treated with curative intent (blue) and non curative intent (red).

Curative surgery was attempted in 17 (43.5%) patients and treatment with non-curative intent, life-prolonging or palliative therapy, was provided to 22 patients (**Figure 2**).

**Figure 3:**
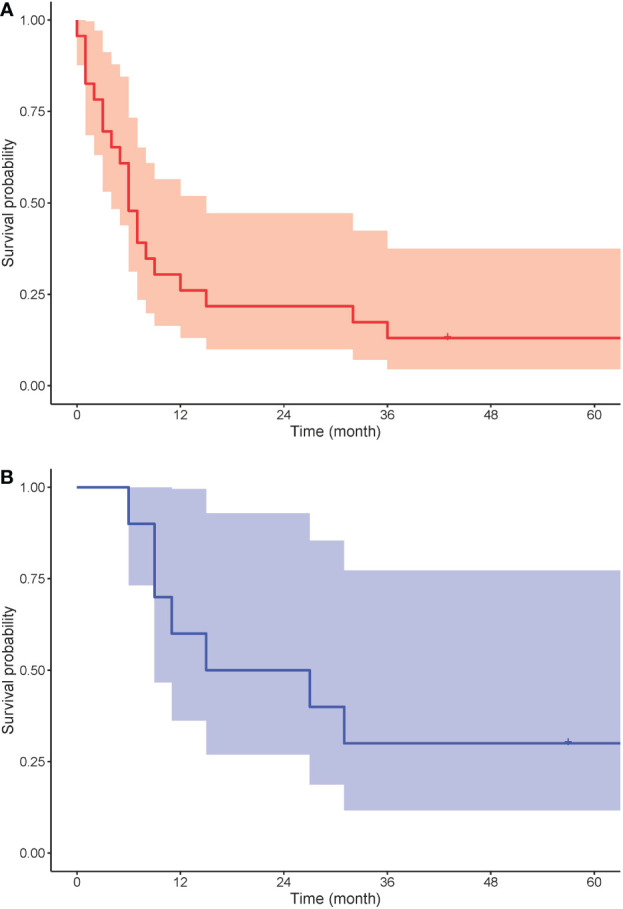
Kaplan-Meier survival analysis for overall survival of **(A)** PDAC (red) and **(B)** BTC (blue).

Ten out of 26 patients with PC underwent surgery with curative intent. Five patients had surgical resection with a negative resection margin. Five patients’ resection margins were positive, and adjuvant chemotherapy was administered to these patients. In LS patients with PC who were treated with curative intent, the 5-year survival rate was 50%. In the 10 LS patients with PDAC resected with curative intent, the 5-year survival rate was 38%. Seven patients were treated with chemotherapy and one patient with chemoradiation therapy. One patient received additional immunotherapy with pembrolizumab for MLH1/PMS2-deficient PDAC. Eight patients were provided with symptomatic treatment. In 16 patients who were not treated with curative intent, the median OS was 5 months. For all patients with PC, the 5-year OS rate was 20%, and for those with PDAC, it was 13.6% (**Figure 3A**). One patient died from pneumonia after a palliative operation within 72 h and was excluded from survival calculations. Endoscopic stent placement was performed in eight cases and percutaneous transhepatic drainage in one.

Four patients with BTC underwent surgical resection with curative intent. Three patients’ resection margin was negative. One patient’s resection margin was positive, and adjuvant chemotherapy was administered. Among these four patients with BTC who were operated on with curative intent, the 5-year survival rate was 75%. Two patients were treated with chemotherapy, and one with chemoradiation therapy. Three patients were treated symptomatically. In six patients who were not treated with curative intent, the median OS was 10 months. For LS patients with BTC, the 5-year OS rate was 30% (**Figure 3B**). Endoscopic stent placement was performed in three cases, and percutaneous transhepatic drainage was placed in four cases.

All three patients with adenocarcinomas of the ampulla of Vater underwent curative surgery with a negative resection margin. Still two patients died of recurrence of ampullary cancer within 1.5 years and one from small bowel cancer.

Thirty-three patients (85%) out of 39 were deceased. For thirty patients (91%), the cause of death was PC, BTC, or ampullary cancer. Two patients died from another LS-associated cancer and one patient died from a stroke. An overview of clinical characteristics and treatment is presented in **Table 2**.

**Table 2:** Overview of clinical characteristics and treatment in LS patients with pancreatic, biliary tract and ampullary cancer.

	Pancreatic cancer N=26		Biliary tract cancer N=10		Ampullary cancer N=3	
**Age median, years *(range)***	64 *(39-81)*		54 *(34-80)*		53 *(49-65)*	
**Sex**						
**M**	11 *(42%)*		8 *(80%)*		1 *(33%)*	
**F**	15 *(58%)*		2 *(20%)*		2 *(67%)*	
**Genes**						
***MLH1***	21		9		3	
***MSH2***	5		0		0	
***MSH6***	0		1		0	
***PMS2***	0		0		0	
**Histology **						
** **	Ductal adenocarcinoma	23 *(82%)*	Cholangiocarcinoma	9 *(90%)*	Adenocarcinoma	3 *(100%)*
** **	NET	5 *(18%)*	Carcinoma of gallbladder	1 *(10%)*		
**Primary site**						
** **	Head	12	Intrahepatic	2	Ampulla of Vater	3
** **	Body	10	Perihilar	5		
** **	Tail	5	Common bile duct	2		
** **	*Not Available*	1	Gallbladder	1		
**Metachronous tumors**						
**Number median *(range)***		1 *(0-3)*		1 *(0-4)*		3 *(0-6)*
**Colorectal cancer**		17		10		5
**Endometrial cancer**		6		1		1
**Treatment **						
**Curative intent**		10 *(38%)*		4 *(40%)*		3 *(100%)*
**Non curative intent**		16 *(62%)*		6 *(60%)*		0
**5-year survival**	20%		30%		33%	

## 4 Discussion

Sporadic PCs and BTCs have a dismal prognosis due to being asymptomatic in the early stages, resulting in a late diagnosis. Treatment options are limited and lack effectiveness. The five-year survival rate is only 10% for both cancer types in the United States (25, 26). LS patients have an increased lifetime risk of developing PC and BTC. Analysis of data from the Prospective Lynch Syndrome Database (PLSD) has shown the cumulative risk at 75 years of age for PC is 6.2%, 0.5%, and 1.4%, and for BTC is 3.7%, 1.7%, and 0%, respectively, for carriers of *MLH1*, *MSH2*, and *MSH6* germline variants (5). Our retrospective study supports pathogenic *MLH1* germline variant carriers being overrepresented among LS patients with PC and BTC.

We did not detect any pancreaticobiliary cancers in *PMS2* carriers, although the number of identified *PMS2* families is low in Finland. No clear evidence of an increased risk of PC or BTC has been shown in *PMS2* pathogenic variant carriers, even in the larger series (27). In a study by Møller et al., none of the 124 *PMS2* pathogenic variant carriers were diagnosed with PC or BTC (5). Hu et al. reported three *PMS2* carriers with PDAC. Two of these patients developed MMR-proficient PDAC (28). Ando et al. performed immunohistochemistry (IHC) analysis on 116 operated BTC patients, identifying two *PMS2* germline variant carriers, both microsatellite stable (MSS) (29). These findings suggest that PDAC and BTC in *PMS2* germline mutation carriers might be sporadic. The cautionary tale of Wang et al. raises the importance of routine tumor testing for both MMR deficiency and MSI to detect patients who might have a better chance of responding to immunotherapy (30). Hendifar et al. presented a case report underscoring the importance of testing every cancer in LS patients for MMR, as not all of them might respond to immunotherapy (31).

The incidence of sporadic PC and BTC increases with age and the median age at the diagnosis is 70 years (32). In the current study, LS patients with PC or BTC were younger, resembling the early age phenomenon which is typical for all LS associated cancers. Also, PC was diagnosed equally in females and males. The small sample size of this study does not allow definitive conclusions drawn, but the latest PLSD report did report substantial sex difference in upper gastrointestinal cancers with 22% in male *MLH1* carriers by 75 years versus 11% in female *MLH1* carriers (33).

Three-quarters of LS patients with PC or BTC had metachronous tumors. Colorectal and endometrial cancers were diagnosed in all cases prior to PC or BTC. These findings suggest that most, but not all, LS patients develop PC or BTC later in life after the more common primary cancers. A personal cancer history of LS carriers over 60 years of age may serve as an indicator for healthcare to stay alert for unspecific symptoms the upper gastrointestinal cancers may induce. On the other hand, a quarter of the patients did not have a previous cancer history, and PC or BTC was their first malignancy.

Most sporadic PCs are in the head of the pancreas. PC in the body or tail has a worse prognosis compared to pancreatic head cancer due to remaining asymptomatic for a longer period, resulting in late diagnoses (34, 35). Takamizawa et al. described the anatomical location of the PC in six LS patients, identifying five of the six PCs as being in the body and the tail of the pancreas (36). In our study, PC was located equally often in the head, the body, and the tail of the pancreas. Our series suggests that no distinct primary anatomical site is more prevalent. BTC was found in all parts of the biliary tract, as also previously shown by Cloyd et al. (11).

Surgical resection is the only curative treatment for PC and BTC. Curative surgery can be performed in 10%–20% of sporadic PC cases and in 20% of sporadic BTC cases (37, 38). In this study, curative surgical resection was performed twice as often, resulting in better overall survival outcomes. This might be explained by the fact that half of the cases already had an LS diagnosis and participated in regular surveillance. In Finland, surveillance for PC and BTC in LS patients is symptom-based, as European guidelines for LS recommend (13). In practice, it means LS patients are educated about symptoms they might encounter and are encouraged to contact secondary and tertiary healthcare providers with expertise in LS if they experience any “red flag” symptoms.

Immune modulation therapy with checkpoint inhibition is a new promising option for LS-associated cancer types with poor prognosis, as histology-agnostic FDA approval for any dMMR or MSI solid cancers is in place (18). Pancreaticobiliary cancers are often deemed unresectable, but a proper molecular pathological examination revealing MSI with even some response to checkpoint inhibition may convert an inoperable case back to operable. However, good biopsies for histology might be difficult to get, and especially known LS carriers should be referred to experienced centers with high volumes of endoscopic ultrasound-guided biopsies to avoid false-negative biopsies for dMMR and MSI due to poor sample quality. It is especially important to not suffice with imaging or cytology-informed diagnosis alone, but a diagnostic biopsy for dMMR or MSI testing must be obtained in all cases with known or suspected LS.

This study has several limitations, such as a retrospective design, a small sample size, and, in some cases, a lack of pathological verification of cancer. Even though the small sample size of this cohort limits the power of statistical analysis, it is still the largest series reported to date. Although it seems that the survival of LS patients with PC or BTC is better than in sporadic cancers, it is still poor. The relatively higher surgical resectability may be accounted for by selection bias due to the earlier age of onset.

To conclude, there is a growing need for molecular and immune profiling of LS-associated PDAC and BTC to clarify the suitability of these cancers with an extremely poor prognosis for immune or any other upcoming therapy.

## Data availability statement

The original contributions presented in the study are included in the article/supplementary materials. Further inquiries can be directed to the corresponding authors.

## Ethics statement

Ethical review and approval was not required for the study on human participants in accordance with the local legislation and institutional requirements. Written informed consent for participation was not required for this study in accordance with the national legislation and the institutional requirements.

## Author contributions

KZ: Data curation, formal analysis, writing—original draft. J-PM: Conceptualization, supervision, writing—review and editing. TS: Supervision, writing—review and editing. All authors contributed to the article and approved the submitted version.

## References

[1] Lynch H Lynch P Lanspa S Snyder C Lynch J Boland C. Review of the lynch syndrome: history, molecular genetics, screening, differential diagnosis, and medicolegal ramifications. Clin Genet (2009) 76(1):1–18. doi: 10.1111/j.1399-0004.2009.01230.x PMC2846640 19659756

[2] Ligtenberg MJL Kuiper RP Chan TL Goossens M Hebeda KM Voorendt M. Heritable somatic methylation and inactivation of MSH2 in families with lynch syndrome due to deletion of the 3′ exons of TACSTD1. Nat Genet (2009) 41(1):112–7. doi: 10.1038/ng.283 19098912

[3] Kovacs ME Papp J Szentirmay Z Otto S Olah E. Deletions removing the last exon of TACSTD1 constitute a distinct class of mutations predisposing to lynch syndrome. Hum Mutation. (2009) 30(2):197–203. doi: 10.1002/humu.20942 19177550

[4] Win AK Jenkins MA Dowty JG Antoniou AC Lee A Giles GG. Prevalence and penetrance of major genes and polygenes for colorectal cancer. Cancer Epidemiol Biomarkers Prev (2017) 26(3):404–12. doi: 10.1158/1055-9965.EPI-16-0693 PMC5336409 27799157

[5] Møller P Seppälä TT Bernstein I Holinski-Feder E Sala P Evans DG. Cancer risk and survival in path_MMR carriers by gene and gender up to 75 years of age: a report from the prospective lynch syndrome database. Gut (2018) 67(7):1306–16. doi: 10.1136/gutjnl-2017-314057 PMC6031262 28754778

[6] Lynch HT Voorhees GJ Lanspa SJ McGreevy PS Lynch JF. Pancreatic carcinoma and hereditary nonpolyposis colorectal cancer: a family study(1985). Available at: https://www.ncbi.nlm.nih.gov/pmc/articles/PMC1977116/ (Accessed April 6, 2022). 10.1038/bjc.1985.187 PMC1977116 4027169

[7] Mecklin JP Järvinen HJ Virolainen M. The association between cholangiocarcinoma and hereditary nonpolyposis colorectal carcinoma. Cancer (1992) 69(5):1112–4. doi: 10.1002/cncr.2820690508 1310886

[8] Kastrinos F Stoffel EM Balmaña J Steyerberg EW Mercado R Syngal S. Phenotype comparison of MLH1 and MSH2 mutation carriers in a cohort of 1,914 individuals undergoing clinical genetic testing in the united states. Cancer Epidemiology Biomarkers Prev (2008) 17(8):2044–51. doi: 10.1158/1055-9965.EPI-08-0301 18708397

[9] Geary J Sasieni P Houlston R Izatt L Eeles R Payne SJ. Gene-related cancer spectrum in families with hereditary non-polyposis colorectal cancer (HNPCC). Familial Cancer. (2008) 7(2):163–72. doi: 10.1007/s10689-007-9164-6 17939062

[10] Borelli I Casalis Cavalchini GC Del Peschio S Micheletti M Venesio T Sarottoet I. A founder MLH1 mutation in lynch syndrome families from piedmont, Italy, is associated with an increased risk of pancreatic tumours and diverse immunohistochemical patterns. Fam Cancer. (2014) 13(3):401–13. doi: 10.1007/s10689-014-9726-3 24802709

[11] Cloyd JM Chun YS Ikoma N Vauthey JN Aloia TA Cuddy A. Clinical and genetic implications of DNA mismatch repair deficiency in biliary tract cancers associated with lynch syndrome. J Gastrointest Cancer. (2018) 49(1):93–6. doi: 10.1007/s12029-017-0040-9 PMC7703856 29238914

[12] Bujanda L Herreros-Villanueva M. Pancreatic cancer in lynch syndrome patients. J Cancer. (2017) 8(18):3667–74. doi: 10.7150/jca.20750 PMC5688919 29151953

[13] Seppälä TT Latchford A Negoi I Sampaio Soares A Jimenez-Rodriguez R Sanchez-Guillén L. European Guidelines from the EHTG and ESCP for lynch syndrome: an updated third edition of the mallorca guidelines based on gene and gender. Br J Surgery. (2021) 108(5):484–98. doi: 10.1002/bjs.11902 PMC10364896 34043773

[14] Crosbie EJ Ryan NAJ Arends MJ Bosse T Burn J Corneset JM. The Manchester international consensus group recommendations for the management of gynecological cancers in lynch syndrome. Genet Med (2019) 21(10):2390–400. doi: 10.1038/s41436-019-0489-y PMC6774998 30918358

[15] Ryan N a. J Evans DG Green K Crosbie EJ. Pathological features and clinical behavior of lynch syndrome-associated ovarian cancer. Gynecologic Oncol (2017) 144(3):491. doi: 10.1016/j.ygyno.2017.01.005 PMC5345899 28065618

[16] Møller P Seppälä T Bernstein I Holinski-Feder E Sala P Evans DG. Cancer incidence and survival in lynch syndrome patients receiving colonoscopic and gynaecological surveillance: first report from the prospective lynch syndrome database. Gut (2017) 66(3):464–72. doi: 10.1136/gutjnl-2015-309675 PMC5534760 26657901

[17] Le DT Durham JN Smith KN Wang H Bartlett BR Aulakh LK. Mismatch-repair deficiency predicts response of solid tumors to PD-1 blockade. Science (2017) 357(6349):409–13. doi: 10.1126/science.aan6733 PMC5576142 28596308

[18] Marcus L Lemery SJ Keegan P Pazdur R. FDA Approval summary: Pembrolizumab for the treatment of microsatellite instability-high solid tumors. Clin Cancer Res (2019) 25(13):3753–8. doi: 10.1158/1078-0432.CCR-18-4070 30787022

[19] Overman MJ McDermott R Leach JL Lonardi S Lenz H-J Morse MA. Nivolumab in patients with metastatic DNA mismatch repair deficient/microsatellite instability–high colorectal cancer (CheckMate 142): results of an open-label, multicentre, phase 2 study. Lancet Oncol (2017) 18(9):1182–91. doi: 10.1016/S1470-2045(17)30422-9 PMC6207072 28734759

[20] Zeng FL Chen JF. Application of immune checkpoint inhibitors in the treatment of cholangiocarcinoma. Technol Cancer Res Treat (2021) 20:15330338211039952. doi: 10.1177/15330338211039952 34528830 PMC8450549

[21] R: The r project for statistical computing . Available at: https://www.r-project.org/ (Accessed May 5, 2022).

[22] Publication of WHO classification of tumours, 5th edition, volume 1: Digestive system tumours . IARC. Available at: https://www.iarc.who.int/news-events/publication-of-who-classification-of-tumours-5th-edition-volume-1-digestive-system-tumours/ (Accessed May 5, 2022).

[23] Khanna L Prasad SR Sunnapwar A Kondapaneni S Dasyam A Tammisetti VS. Pancreatic neuroendocrine neoplasms: 2020 update on pathologic and imaging findings and classification. RadioGraphics (2020) 40(5):1240–62. doi: 10.1148/rg.2020200025 32795239

[24] Kendall T Verheij J Gaudio E Evert M Guido M Goeppert B. Anatomical, histomorphological and molecular classification of cholangiocarcinoma. Liver Int (2019) 39(S1):7–18. doi: 10.1111/liv.14093 30882996

[25] Pancreatic cancer - statistics (2012). Cancer.Net. Available at: https://www.cancer.net/cancer-types/pancreatic-cancer/statistics (Accessed October 20, 2022).

[26] Bile duct cancer (Cholangiocarcinoma) - statistics (2012). Cancer.Net. Available at: https://www.cancer.net/cancer-types/bile-duct-cancer-cholangiocarcinoma/statistics (Accessed October 20, 2022).

[27] ten Broeke SW van der Klift HM Tops CMJ Aretz S Bernstein I Buchanan DD. Cancer risks for PMS2-associated lynch syndrome. JCO (2018) 36(29):2961–8. doi: 10.1200/JCO.2018.78.4777 PMC6349460 30161022

[28] Hu ZI Shia J Stadler ZK Varghese AM Capanu M Salo-Mullen E. Evaluating mismatch repair deficiency in pancreatic adenocarcinoma: Challenges and recommendations. Clin Cancer Res (2018) 24(6):1326–36. doi: 10.1158/1078-0432.CCR-17-3099 PMC5856632 29367431

[29] Ando Y Kumamoto K Matsukawa H RYOU Ishikawa R Suto H Oshima M. Low prevalence of biliary tract cancer with defective mismatch repair genes in a Japanese hospital-based population. Oncol Lett (2022) 23(1):4. doi: 10.3892/ol.2021.13122 34820003 PMC8607234

[30] Wang Y Cuggia A Pacis A Boileau J-C Marcuset VA Gao ZH. Pancreatic cancer progression in a patient with lynch syndrome receiving immunotherapy: A cautionary tale. J Natl Compr Cancer Network. (2021) 19(8):883–7. doi: 10.6004/jnccn.2021.7049 34416708

[31] Hendifar AE Larson BK Rojansky R Guan M Gong J Veronica Placencio V. Pancreatic cancer ‘mismatch’ in lynch syndrome. BMJ Open Gastroenterol (2019) 6(1):e000274. doi: 10.1136/bmjgast-2019-000274 PMC6577306 31275582

[32] Wang H Liu J Xia G Lei S Huang X Huang X. Survival of pancreatic cancer patients is negatively correlated with age at diagnosis: a population-based retrospective study. Sci Rep (2020) 10(1):7048. doi: 10.1038/s41598-020-64068-3 32341400 PMC7184604

[33] Dominguez-Valentin M Sampson JR Seppälä TT ten Broeke SW Plazzer J-P Nakken S. Cancer risks by gene, age, and gender in 6350 carriers of pathogenic mismatch repair variants: findings from the prospective lynch syndrome database. Genet Med (2020) 22(1):15–25. doi: 10.1038/s41436-019-0596-9 31337882 PMC7371626

[34] Artinyan A Soriano PA Prendergast C Low T Ellenhorn JDI Kim J. The anatomic location of pancreatic cancer is a prognostic factor for survival. HPB (Oxford). (2008) 10(5):371–6. doi: 10.1080/13651820802291233 PMC2575681 18982154

[35] Lee M Kwon W Kim H Byun Y Han Y Kanget JS. The role of location of tumor in the prognosis of the pancreatic cancer. Cancers (Basel). (2020) 12(8):2036. doi: 10.3390/cancers12082036 32722142 PMC7465041

[36] Takamizawa S Morizane C Tanabe N Maruki Y Kondo S Susumu Hijioka S. Clinical characteristics of pancreatic and biliary tract cancers associated with lynch syndrome. J Hepatobiliary Pancreat Sci (2022) 29(3):377–84. doi: 10.1002/jhbp.1063 PMC9298874 34689428

[37] Cillo U Fondevila C Donadon M Gringeri E Mocchegiani F Schlitt HJ. Surgery for cholangiocarcinoma. Liver Int (2019) 39(Suppl 1):143–55. doi: 10.1111/liv.14089 PMC6563077 30843343

[38] Vincent A Herman J Schulick R Hruban RH Goggins M. Pancreatic cancer. Lancet (2011) 378(9791):607–20. doi: 10.1016/S0140-6736(10)62307-0 PMC3062508 21620466

